# Galectin-9/TIM-3 Interaction Regulates Virus-Specific Primary and Memory CD8^+^ T Cell Response

**DOI:** 10.1371/journal.ppat.1000882

**Published:** 2010-05-06

**Authors:** Sharvan Sehrawat, Pradeep B. J. Reddy, Naveen Rajasagi, Amol Suryawanshi, Mitsuomi Hirashima, Barry T. Rouse

**Affiliations:** 1 Department of Pathobiology, College of Veterinary Medicine, University of Tennessee, Knoxville, Tennessee, United States of America; 2 Department of Immunology & Immunopathology, Faculty of Medicine, Kagawa University, Kagawa, Japan; Oregon Health Sciences University, United States of America

## Abstract

In this communication, we demonstrate that galectin (Gal)-9 acts to constrain CD8^+^ T cell immunity to Herpes Simplex Virus (HSV) infection. In support of this, we show that animals unable to produce Gal-9, because of gene knockout, develop acute and memory responses to HSV that are of greater magnitude and better quality than those that occur in normal infected animals. Interestingly, infusion of normal infected mice with α-lactose, the sugar that binds to the carbohydrate-binding domain of Gal-9 limiting its engagement of T cell immunoglobulin and mucin (TIM-3) receptors, also caused a more elevated and higher quality CD8^+^ T cell response to HSV particularly in the acute phase. Such sugar treated infected mice also had expanded populations of effector as well as memory CD8^+^ T cells. The increased effector T cell responses led to significantly more efficient virus control. The mechanisms responsible for the outcome of the Gal-9/TIM-3 interaction in normal infected mice involved direct inhibitory effects on TIM-3^+^ CD8^+^ T effector cells as well as the promotion of Foxp3^+^ regulatory T cell activity. Our results indicate that manipulating galectin signals, as can be achieved using appropriate sugars, may represent a convenient and inexpensive approach to enhance acute and memory responses to a virus infection.

## Author Summary

Adaptive immune responses to foreign antigens require precise regulation, otherwise excessive bystander damage to host tissues may occur and responses to other antigens could be compromised. Some galectin proteins binding to receptors on cells of the immune system form part of the regulatory system, although this topic has received scant attention as it relates to antiviral control. In this report, we evaluate the role of the galectin-9/TIM-3 receptor pathway at regulating acute and memory CD8^+^ T cell responses to herpes simplex virus (HSV) infection. We demonstrate that CD8^+^ T cell responses to HSV were significantly increased in magnitude and improved in quality in mice unable to produce galectin-9 because of gene knockout compared to wild type controls. Furthermore, inhibiting the galectin-9 mediated response to TIM-3 using the sugar alpha-lactose that binds to Gal-9 led to a similar response pattern. The influence of galectin-9 to constrain CD8^+^ T cell responses involved direct inhibitory effects on the T effectors as well as the promotion of regulatory T cell responses. Our results indicate that manipulating the interaction of galectins with receptors using simple sugars may represent a convenient and inexpensive approach to enhance T cell responses to virus infections, and could prove useful to increase the efficacy of some vaccines.

## Introduction

Adaptive immune responses to foreign antigens require precise regulation. If not, excessive bystander damage to host tissues may occur and an unlimited reaction could erode the size of the repertoire, limiting responses to other antigens. It is evident that the host possesses several mechanisms that control the size, composition and duration of immune reactions [1]. In consequence, after the primary response most cells die leaving a memory population that represents a fraction of the cells that responded initially to the antigen. Moreover, these memory cells rarely account for >10% of the total antigen reactive repertoire [2]. In some circumstances, it would be desirable to expand the size of the memory population and perhaps extend the durability of effector cell activity, since this could improve immunity to certain pathogens. HIV is such an example [3]. Accordingly, understanding how immune responses are constrained could provide clues to reverse the process and improve the efficacy of vaccines.

One family of host proteins that plays multiple roles in innate and adaptive immunity is the galectin proteins. These glycan binding proteins either form lattices on cell surfaces or react with specific receptors and trigger a variety of responses that include apoptosis and changes of cell function [4]. Some galectins bind to the surface of pathogens and this may influence pathogen infectivity and survival [5]. For instance, binding of Gal-1 to HIV increases infectivity of the virus for macrophages [5], [6]. At least two family members, Gal-1 and Gal-9, may play an effective role in terminating the acute inflammatory response as well as restricting the extent of chronic lesions in autoimmune and allergic reactions [4], [7]. Recently, we showed that chronic inflammatory reactions caused by HSV infection of the eye was limited by Gal-9 binding to its specific receptor TIM-3 [8]. This interaction led to inhibitory effects on effector T cells, as well as the expansion of regulatory T (Treg) cell activity.

Currently, it is not clear what role galectins play at regulating acute immune responses to virus infections and whether manipulating galectin binding to their receptors can influence the magnitude and effectiveness of anti-viral immunity. In this communication, we investigate the influence of Gal-9 binding to its receptor TIM-3 on the size and quality of CD8^+^ T cell mediated immunity to a virus infection. We demonstrate that Gal-9 acts to limit the extent of CD8^+^ T cell immunity to HSV infection. In support of this, we show that animals unable to produce Gal-9, because of gene knockout, develop acute and memory responses to HSV that are of greater magnitude and better quality than those that occur in normal infected animals. We also make what we believe is the novel observation that infusion of normal infected mice with α-lactose, the sugar that binds to the carbohydrate-binding domain of Gal-9 limiting its TIM-3 receptor engagement [9], also caused a more elevated and higher quality CD8^+^ T cell response to HSV. Such sugar treated infected mice also had expanded populations of memory CD8^+^ T cells. The mechanisms responsible for the outcome of the Gal-9/TIM-3 interaction in normal infected mice involved both inhibitory effects on TIM-3^+^ T effector cells, as well as the promotion of Foxp3^+^ regulatory T cell activity. Our results indicate that manipulating galectin signals, as can be achieved using appropriate sugars, may represent a convenient and inexpensive approach to enhance acute and memory responses to infectious agents and might be useful to improve responses to some vaccines.

## Materials and Methods

### Ethics statement

All animals were housed in Association for Assessment and Accreditation of Laboratory Animal Care (AAALAC)-approved animal facilities. Institutional Animal Care and Use Committee (IACUC), The University of Tennessee, Knoxville approved all experimental protocols and experiments were performed adhering to protocols created by the committee.

### Mice and virus

Female 5–6-wk-old C57BL/6 and congenic Thy1.1^+^ B6.PL (H-2^b^) mice were purchased from Harlan Sprague-Dawley and Jackson Laboratory, respectively and housed in the animal facilities at the University of Tennessee, Knoxville. Galectin-9 KO (Gal-9 KO) mice were kindly provided by GalPharma Co. Ltd, Japan. Dr. Thandi Onami kindly provided P14 LCMV transgenic mice. Foxp3-GFP knock-in mice were kindly provided by Dr. Mohammed Oukka of Harvard Medical School. HSV-1 strain KOS was grown in Vero cells obtained from American Type Culture Collection. The harvested virus was titrated and stored in aliquots at −80°C until further use.

### Antibodies and reagents

Conjugated antibodies purchased from BD Bioscience were anti-CD8α, anti-CD25c anti-CD62 ligand (CD62L), anti-CD44, anti-CD69, anti-IFN-γ, anti-TNF-α, anti-IL-2, anti-CD25 (7D4), anti-KLRG1, anti-CD80, anti-CD86, anti-CD11b, anti-CD11c, anti-CD40, anti-MHC class II and anti-CD103. PE and APC conjugated TIM-3 antibodies were purchased from R&D Systems. Intra-nuclear Foxp3 staining was performed using a kit from ebioscience according to the instructions. Carboxyfluorescein Succinimidyl ester (CFSE) was obtained from Molecular Probes. HSV gB_498–505_ peptide (SSIEFARL) was supplied by Genescript. PE- and APC-Kb-gB tetramer was kindly provided by NIH tetramer core facility, Emory University, Atlanta, GA. Recombinant galectin-9 and anti-galectin-9 antibodies (clone1A2 and 108A2) were provided by Gal Pharma, Japan. Recombinant galectin-3 was obtained from R & D systems. α-Lactose was purchased from Acros organics (Geel, Belgium). The antibody-stained cells were acquired with a FACS Calibur (BD Biosciences) and the data were analyzed using the FlowJo software (Tree Star, OR). Sorting of cells was performed by using a FACS Vantage system.

### Infection of mice, treatment with galectin-9 and α-lactose

Mice were infected in footpad (FP) with 2.5×10^5^ PFU of HSV KOS in 30 µl volume. For memory recall response studies, animals were challenged with the same amount of virus in the footpads after a period of at least 30 days. For primary responses, infected animals were divided randomly into different groups. Mice in one group were treated ip with 125 µg of Gal-9 twice daily starting from day 3 until day 5 post infection (p.i). Similarly animals in other groups were treated twice daily starting from day 3 until day 5 with various doses (27 mM, 137 mM, 277 mM and 416 mM) of α-lactose solution made in PBS. For some memory experiments, animals were provided with 277 mM of α-lactose solution in drinking water for 10 days. 12 hrs after the last treatment animals were sacrificed and their isolated lymphoid organs were made into single cell suspension for flow cytometric analysis.

### Quantification of HSV-1 in foot pad tissues

The quantification of HSV-1 in footpad tissue was done as previously reported [10]. Briefly, the mice were sacrificed at the indicated time p.i., the footpad surface was cleaned with 70% isopropyl alcohol, cut and were stored in RPMI without serum and 2-mercaptoethanol at -80°C until use. Tissues were disrupted by chopping with scissors and homogenized by using a homogenizer (Pellet Pestle mortar; Kontes) and centrifuged. The supernatant was used to assess viral titers on Vero cells. Finally, plaques were visualized after staining with crystal violet.

### Adoptive transfer of cells

Different numbers of cells obtained from pooled spleen and Lymph nodes (LN) of Thy1.1 LCMV (P14) transgenic animals were transferred into Thy1.2 C57BL/6 animals that were then infected with HSV-KOS in footpads. At 5dpi, LCMVgp33-Tet^+^ and Kb-gB-Tet^+^ cells were analyzed for the expression of TIM-3. The control animals not transferred with any cell type were similarly infected with HSV KOS. Similarly, for some other experiments, cells obtained from pooled LNs and spleens of B6 Thy1.1 animals were labeled with 2.5 µM of CFSE. 10^7^ of these cells were transferred into B6 Thy1.2 wild type and galectin-9 knockout animals. Alternatively, CD8^+^ T cells obtained from Gal-9 KO and WT animals (both Thy1.2) were CFSE labeled and transferred into Thy1.1 C57BL/6 animals. After 24 hrs of transfer, recipient animals were infected with 2.5×10^5^ PFU of HSV 1 KOS. After five days of infection LNs and spleens were isolated and processed to make single cell suspensions. The dilution of CFSE was analyzed flow cytometrically in Thy1.1^+^ or Thy1.2^+^CD8^+^ T cells.

### SSIEFARL-specific CD8^+^ T cell proliferation

CD8^+^ T cell proliferation was evaluated after in vitro stimulation of splenocytes and draining LN cells obtained from HSV infected mice with MHC class I (H-2K^b^)–restricted SSIEFARL peptide. Briefly, the cells were stimulated with SSIEFARL peptide (1 µg/ml) for 3 days in the presence of 100 U/ml of IL-2. [^3^H] Thymidine (1 µCi/well) was added to each well 18 h before harvest. Harvested cells were measured for radioactivity using a β-scintillation counter (Inotech).

### In vivo CTL assay

The in vivo CTL assay was done as reported earlier [11]. Splenocytes from naive B6 mice were used as target cells and split into two equal populations. One was pulsed with 2.5 µg gB_498–505_ peptide for 45 min at 37°C and then labeled with a high concentration (2.5 µm) of CFSE. The other population was un-pulsed and labeled with a low concentration of CFSE (0.25 µm). Equal numbers of cells from each population (10^7^) were mixed together and adoptively transferred i.v. into naïve and HSV-1–infected mice. Splenocytes and peripheral blood were collected 2 h after adoptive transfer from recipient mice, erythrocytes were lysed, and cell suspensions were analyzed by FACS Calibur system. Each population was distinguished by their respective fluorescence intensity. Assuming that the number of peptide-pulsed cells injected is equivalent to the number of no peptide-pulsed cells injected, the percentage of killing of target cells in uninfected animals was determined as:
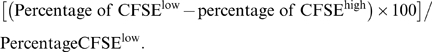



The percentage killing of target cells in infected animals was calculated as:
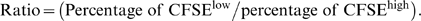


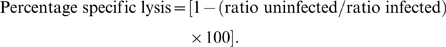



### Intracellular Cytokine Staining Assay (ICCS)

To enumerate the number of IFN-γ, TNF-α and IL-2 producing T cells, intracellular cytokine staining was performed as previously described [12]. In brief, 10^6^ freshly isolated splenocytes were cultured in U bottom 96-well plates. Cells were left untreated or stimulated with SSIEFARL peptide (2 µg/ml), and incubated for 5 h at 37°Cin 5% CO_2_. Brefeldin A (10 µg/ml) was added for the duration of the culture period to facilitate intracellular cytokine accumulation. After this period, cell surface staining was performed, followed by intracellular cytokine staining using a Cytofix/Cytoperm kit (BD PharMingen) as per the manufacturer's recommendations. The Ab used were anti–IFN-γ (clone XMG1.2), anti-TNF-α (clone MP6-XT22), and anti-IL-2 (clone JES6-5H4). The fixed cells were re-suspended in FACS buffer (PBS with 3% heat inactivated serum) and analyzed flow cytometrically.

### Ex vivo apoptosis assay

PLNs single cell suspension isolated 6 days pi from HSV infected animals were incubated for 5 hrs with varying concentrations of Gal-9 or Gal-3 in the absence or the presence of α-lactose in 96-well flat-bottom plates in humidified incubators in the presence of 5% CO_2_. After the incubation period was over, cells were stained for annexin V using a kit from BD Biosciences. Additionally, cells were also co-stained for CD8, TIM-3, Kb-gB-Tet. Stained cells were analyzed immediately by flow cytometry.

### In vitro suppression assay

At 6 dpi, Foxp3^+^CD4^+^ T cells were FACS sorted on a FACS Vantage system to the extent of 97.8% purity from HSV infected Foxp3-GFP-Knock-In animals that were either untreated or treated with 200 µl of 277 mM α-lactose i.p. from day 3 to day 5. Foxp3^+^ T cells were then cultured with anti-CD3 stimulated CFSE labeled CD4^+^CD25^−^ Thy1.1 (MACS purified) and T cell depleted irradiated splenocytes for three days. Thereafter, the extent of CFSE dilution in Thy1.1^+^CD4^+^ T cells was analyzed flow cytometrically.

### Immunoblotting and ELISA for the detection of galectin-9 expression and quantification

Lymph node and spleen homogenate samples (10 µg/lane) were resolved on 15% SDS-PAGE and transferred electrophoretically on to a PVDF membrane (Bio-Rad). The membrane was blocked overnight with 5% BSA and washed 5 times with TBS containing 0.05% Tween-20 (TBST) and incubated with biotinylated anti-galectin antibody (R&D Systems) at a concentration of 0.5 µg/ml diluted in TBST for 1 hr at room temperature. The membrane was washed 5 times with TBST and incubated with streptavidin-HRP antibody (Pierce) at a dilution of 1∶10000 for 1 hr at room temperature. The membrane was developed with chemiluminescent substrate (Immobilon western chemiluminescent HRP substrate, Millipore) and the image was taken on CL-XPosure X-ray film (Thermo scientific).

Ninety-six-well microplates were coated with capture antibody at a concentration of 3000 ng/ml (100 µl/well, anti-galectin-9, GalPharma Co. Ltd, Japan). After incubation overnight at 4°C, the wells were washed three times with PBST (PBS containing 0.05% Tween-20) and blocked with 5% BSA for 2 h at RT. The wells were washed three times with PBST and Lymph node, spleen homogenate samples (100 µl) were added to the wells and incubated at RT for 2 h and aspirated. The wells were then washed with wash buffer. Biotinylated anti galectin detection antibody (1;10000, R&D Systems) diluted in reagent diluent (R&D Systems, PBS, 5% Tween 20, 2% goat serum) was added to each well and incubated at RT for 1 h. The wells were then washed three times and 100 µl of streptavidin–horseradish peroxidase (1∶10000 dilution) added and incubated for 1 hr at room temperature. The plate was washed thrice and developed with TMB (R&D systems) substrate and the absorbance of each sample was determined at 450 nm. A standard curve ranging from 5 µg to 156.25 ng of recombinant galectin-9 (Gal Pharma, Japan) was generated to calculate the galectin-9 concentration in the unknown samples.

### Statistical analysis

Most of the analyses for determining the level of significance were performed using Student's t test by Graphpad software. Values of p 0.001 (***), p 0.01 (**), and p 0.05 (*) were considered significant. Results are expressed as mean ± SEM or otherwise stated.

## Results

### Virus-specific CD8^+^ T cells up regulate TIM-3 expression after HSV infection

**Figure 1:**
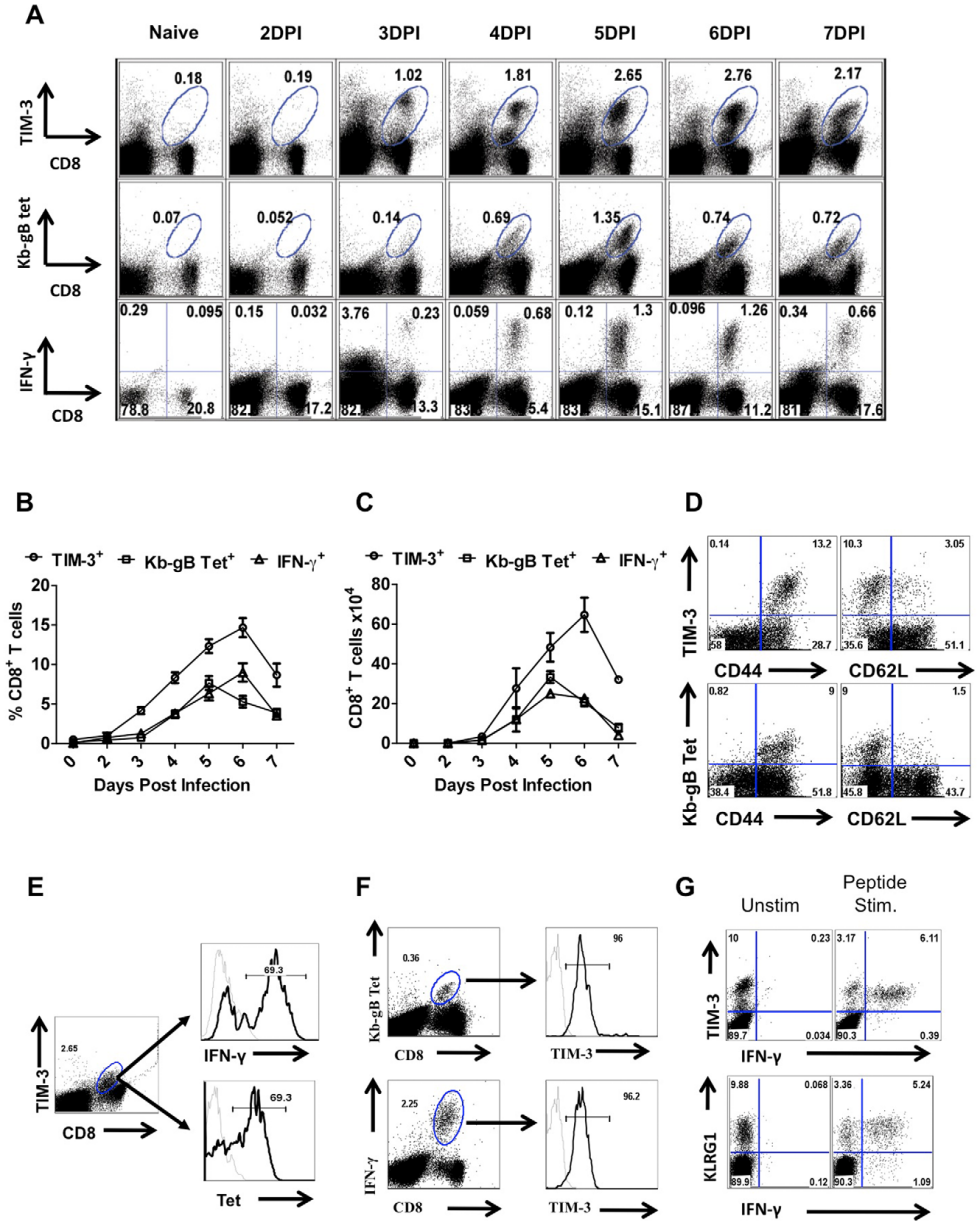
TIM-3 expression is up regulated on virus-specific CD8^+^ T cells after HSV infection. C57BL/6 animals were infected in each hind footpad with 2.5×10^5^ PFU of HSV. At different time points after infection, draining popliteal LNs (PLNs) cells isolated from three animals at each time point were analyzed flow cytometrically for TIM-3, Kb-gB-tetramer and IFN-γ staining. A. FACS plots showing the frequencies of TIM-3^+^ (upper panel), Kb-gB-Tet^+^ (middle panel) and SSIEFRAL-peptide stimulated IFN-γ producing (lower panel) CD8^+^ T cells are shown. Percentages (B) and absolute numbers (C) of TIM-3^+^, Kb-gB-Tet^+^ and SSIEFRAL-peptide stimulated IFN-γ producing CD8^+^ T cells in the draining PLN of HSV infected animals are shown. D. Co-expression of TIM-3 (upper panel) and Kb-gB-Tet (lower panel) with CD44 and CD62L is shown by representative FACS plots. E. FACS plots showing IFN-γ production by TIM-3^+^ (upper panel) and Kb-gB-Tet^+^ expression by TIM-3^+^CD8^+^ T cells are shown. F. Representative FACS plots show TIM-3 expression on Kb-gB-Tet^+^ (upper panel) and IFN-γ^+^CD8^+^ T (lower panel) cells G. FACS plots show the expression of TIM-3 (upper panel) and KLRG1 (lower panel) on IFN-γ producing CD8^+^ T cells.

CD8^+^ T cells isolated from the draining popliteal LNs (PLN) and spleens of HSV infected animals were analyzed for TIM-3 expression at different times pi. As is evident in Fig 1A, whereas few CD8^+^ T cells isolated from LNs of uninfected animals expressed TIM-3, after HSV infection TIM-3^+^ cells were numerous. This increase in TIM-3^+^CD8^+^ T cells was evident at day 3 pi in the PLN and day 4 in the spleen (Figure S1) and peak frequencies (Fig 1A and B), as well as total numbers (Fig 1C), were present on days 6. The majority of TIM-3^+^ T cells isolated from infected animals were CD44^hi^ and CD62L^lo^ suggesting that only activated or effector T cells expressed surface TIM-3^+^ (Fig 1D).

**Figure 2:**
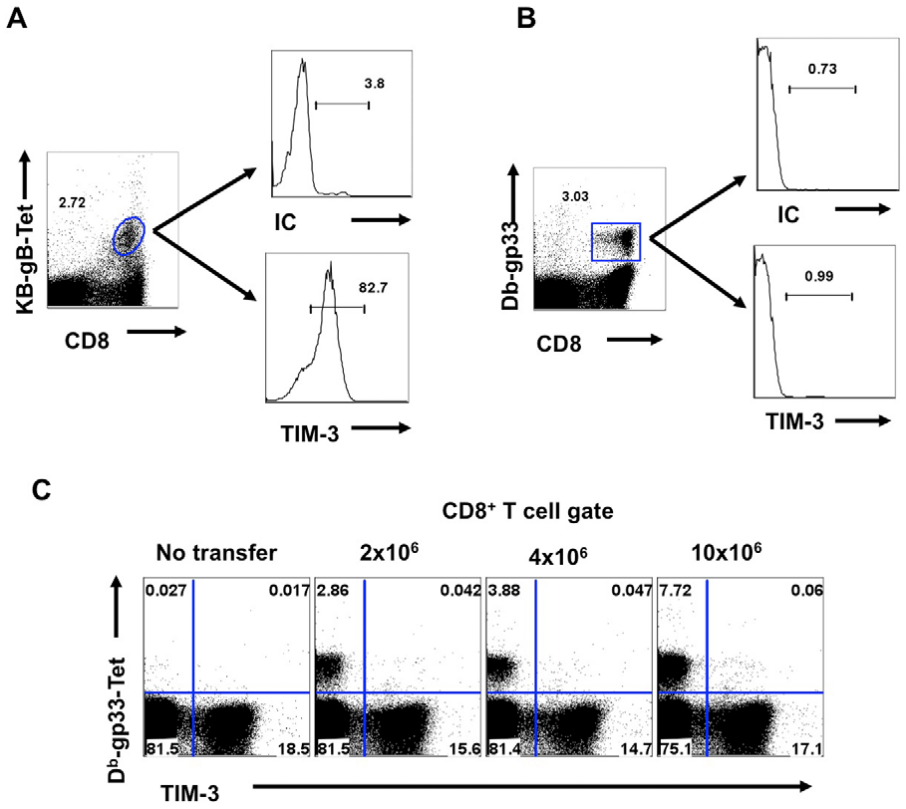
TIM-3 is expressed on TCR stimulated CD8^+^ T cells. C57BL/6 animals were injected i.v. with varying numbers of splenocytes isolated from P14 transgenic animals (Thy1.1) before infection with HSV. 5 days pi PLN and spleens were isolated and analyzed for the expression of TIM-3 on Kb-gB-Tet^+^ and Db-gp33-41-Tet^+^ CD8^+^ T cells. A-B. FACS plots showing the expression of TIM-3 on Kb-gb-Tet^+^ (A) and Db-gp33-41-Tet^+^ (B) isolated from PLN. C. Expression of TIM-3 on endogenous and donor Db-gp33-41-Tet^+^CD8^+^ T cells when varying numbers of P 14 cells were transferred.

Using tetramers and the ICCS assay to detect SSIEFARL specific CD8^+^ T cells (this represents the immunodominant response to HSV in C57BL6 mice [13], almost all cells were TIM-3^+^ at the peak response time (Fig 1E and F). Additionally, the frequency of IFN-γ-producing peptide-specific T cells was comparable for both KLRG1^+^ and TIM-3^+^ cells, further indicating that TIM-3 is a marker for effector cells (Fig 1G). The additional TIM-3^+^CD8^+^ T cells that were not SSIEFARL specific would likely be recognizing other HSV antigens, but this could not be verified for lack of additional reagents. However, it was also conceivable that some of the TIM-3^+^ cells were not HSV specific and represented a bystander-activated population, which has been advocated to occur in some HSV-induced immunopathological lesions [14]. To address the involvement of bystander activation different numbers of P14 transgenic CD8^+^ T cells (these recognize the gp33 peptide of LCMV [15] and are not known to show any cross-reactivity with HSV), were transferred into B6 animals that were subsequently footpad infected with HSV. After 5 days, the expression of TIM-3 was analyzed on Thy1.1^+^gp33-tetramer^+^ and Kb-gB Tet^+^ CD8^+^ T cells. As is evident from Fig 2A-C, we failed to detect any P14 T cells that expressed TIM-3 on their surface, but in the same animals almost all of the responding HSV specific cells detected by the Kb-gB-tetramer were TIM-3^+^. These data add no support for bystander mechanisms causing TIM-3 expression on CD8^+^ T cells. It likely means that all TIM-3^+^CD8^+^ T cells were reacting with viral antigens, but this needs to be formally shown.

### Galectin-9 induces apoptosis of CD8^+^ T cells in vitro

**Figure 3:**
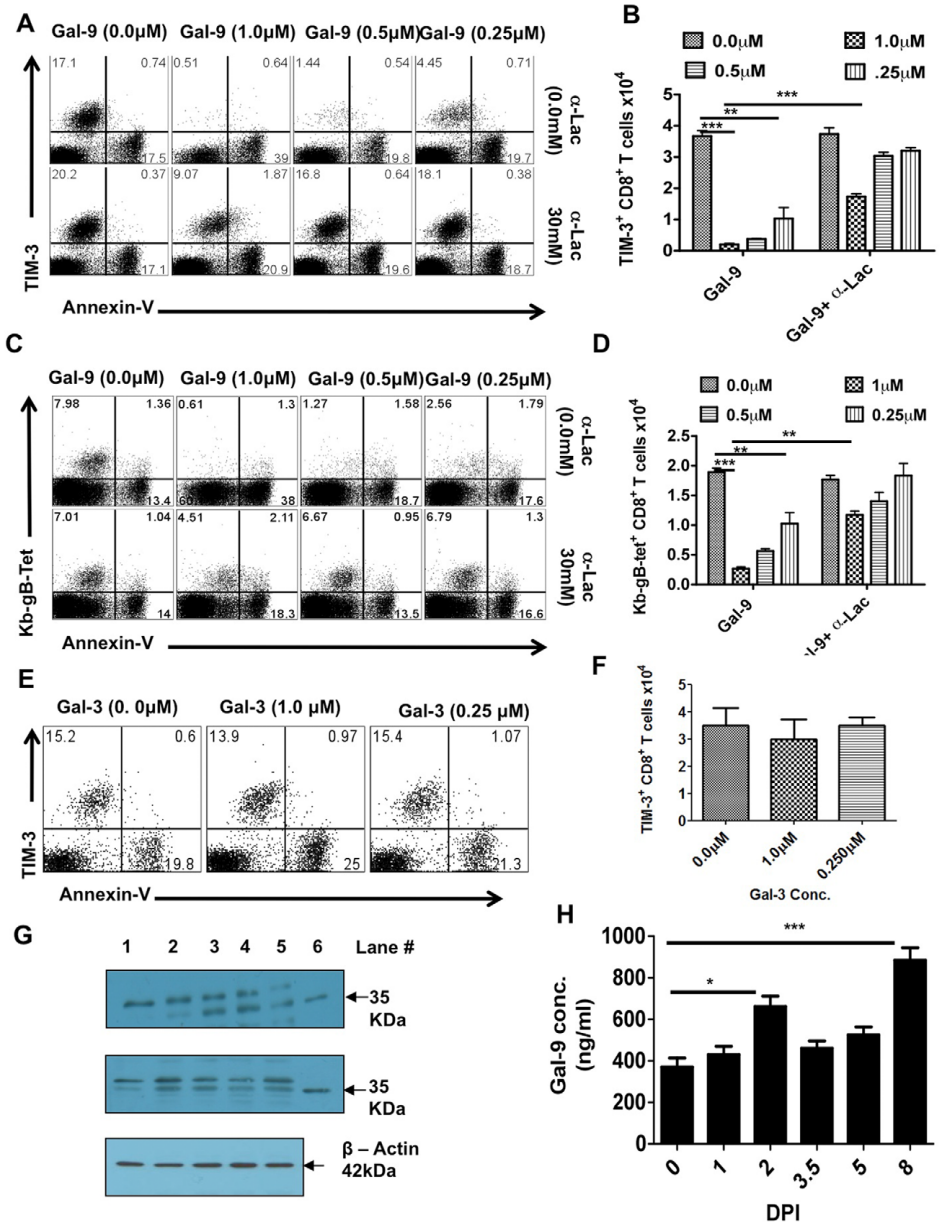
Galectin-9 induces apoptosis of TIM-3 expressing CD8^+^ T cells in vitro and its expression is up regulated in the lymphoid organs after HSV infection. PLNs single cell suspension isolated 6 dpi from HSV infected animals were incubated for 5 hr with varying concentrations of galectin-9 in the absence or the presence of α-lactose. The experiments were repeated multiple times with similar results. A. Representative FACS plots showing the expression of TIM-3 and annexin-V on gated CD8^+^ T cells under indicated incubation conditions are shown. B. The bar diagram shows the numbers of TIM-3^+^CD8^+^ T cells as calculated from A. C. Representative FACS plots showing the expression of Kb-gB-Tet and annexin-V on gated CD8^+^ T cells under indicated incubation conditions are shown. D. The bar diagram shows the numbers of TKb-gB-Tet^+^CD8+ T cells as calculated from C. E. TIM-3 and annexin-V expression on gated CD8^+^ T cells isolated from PLNs of HSV infected mice at 6 dpi and incubated for 5 hr with different concentrations of galectin-3 is shown. F. The bar diagram shows the numbers of TIM-3^+^CD8^+^ T cells as calculated from E. G. Immunoblots showing galectin-9 expression in the homogenates of isolated PLNs (upper panel) and spleens (middle panel) obtained from HSV infected animals at different time points are shown. Expression of β-actin as sample loading control is shown in the bottom panel. (lane # 1, day 0; #2, day 2, #3, day 3.5, #4, day 5, #5, day 8 and #6, recombinant galectin-9). H. Galectin-9 concentrations as measured by sandwich ELISA using anti-Gal-9 mAb (1A2) in the PLN homogenates is shown. The experiments were performed three times and three animals were sacrificed at each time point.

The results of previous in vitro studies have revealed that Gal-9 binding to TIM-3 receptors on some, although not all, T cell subsets causes them to undergo apoptosis [8], [16]. To test the fate of the CD8^+^TIM3^+^ population expanded by HSV infection to Gal-9 exposure, PLN cells were collected 6 days pi and exposed in vitro for 5 hrs to a range of concentrations of Gal-9. Subsequently, the cells were analyzed by FACS for changes in the expression levels of TIM-3 and annexin V, the latter indicative of apoptosis [17]. As shown in Fig 3A, approximately 15% of CD8^+^ T cells were TIM-3^+^ at the onset of culture and this percentage did not change significantly in the absence of Gal-9. However, Gal-9 addition (at1.0 µM) caused a loss of almost all cells that were TIM-3^+^ (Fig 3A upper panel). Baseline levels of annexin V^+^ cells also did not change significantly in the absence of Gal-9 (or in the presence of Gal-3, as shown in Fig 3E-F). However, when optimal amounts of Gal-9 were present, annexin V^+^ cells increased 15–20% beyond baseline numbers (Fig 3A upper panel and 3B). This number roughly correlated with the fraction of TIM-3^+^CD8^+^ T cells that disappeared upon Gal-9 exposure. Under the conditions used, we failed to detect significant numbers of TIM-3^+^ annexin V^+^ cells, although at earlier time points some such cells can be demonstrated (data not shown). In additional experiments, we measured the effects of Gal-9 addition on the fate of Tet^+^ TIM-3^+^ CD8^+^ T cells, almost all of which as described previously were TIM-3^+^. As shown in Fig 3C (upper panel) and 3D, the great majority of Tet^+^ TIM-3^+^ T cells were lost after Gal-9 exposure and there was a large increase in annexin V^+^ T cells.

In other experiments with Gal-9 treated cultures, we investigated the effects of adding an excess of α-lactose, the sugar that binds to the carbohydrate binding domain of Gal-9 and reduces Gal-9 binding to TIM-3 [9], [16]. In such experiments, lactose addition served to prevent the disappearance of most TIM-3^+^ as well as Tet^+^CD8^+^ T cells and also reduced the increase in annexin V^+^ cells (Fig 3A, lower panel and 3B as well as 3C, lower panel and 3D). Taken together, our results imply that Gal-9 binding to TIM-3^+^ effectors caused their death by apoptosis. It is also conceivable that binding of Gal-9 to TIM-3 masks its detection or causes TIM-3 down regulation, but such effects have not been noted in other systems.

### Galectin-9 is up regulated in the lymphoid tissues responding to HSV infection

The observation that most of the CD8^+^ T cells expanded by HSV infection express TIM-3, along with in vitro observations that exposure to Gal-9 caused the deletion of TIM-3^+^CD8^+^ T cells, could indicate that endogenously produced Gal-9 acts likewise and serves to limit the magnitude of the anti-HSV CD8^+^ T cell response. In consequence, we determined if endogenous levels of Gal-9 could be demonstrated in lymphoid extracts of HSV infected animals, as well as to measure if these levels changed at different time points after infection. The quantification of Gal-9 was done by both western blotting and ELISA (Fig 3H and I and Figure S2). Basal levels of Gal-9 were detectable in control lymphoid extracts and levels were moderately increased 2 days p.i. A greater increase (2–2.5 fold) was also observed in 7 days pi samples (Fig 3H). Accordingly, a consequence of HSV infection is an increase in endogenous Gal-9 levels, particularly around the peak time of CD8^+^ T cell responses.

### Animals unable to produce Gal-9 mount better acute phase virus-specific CD8^+^ T cell responses

**Figure 4:**
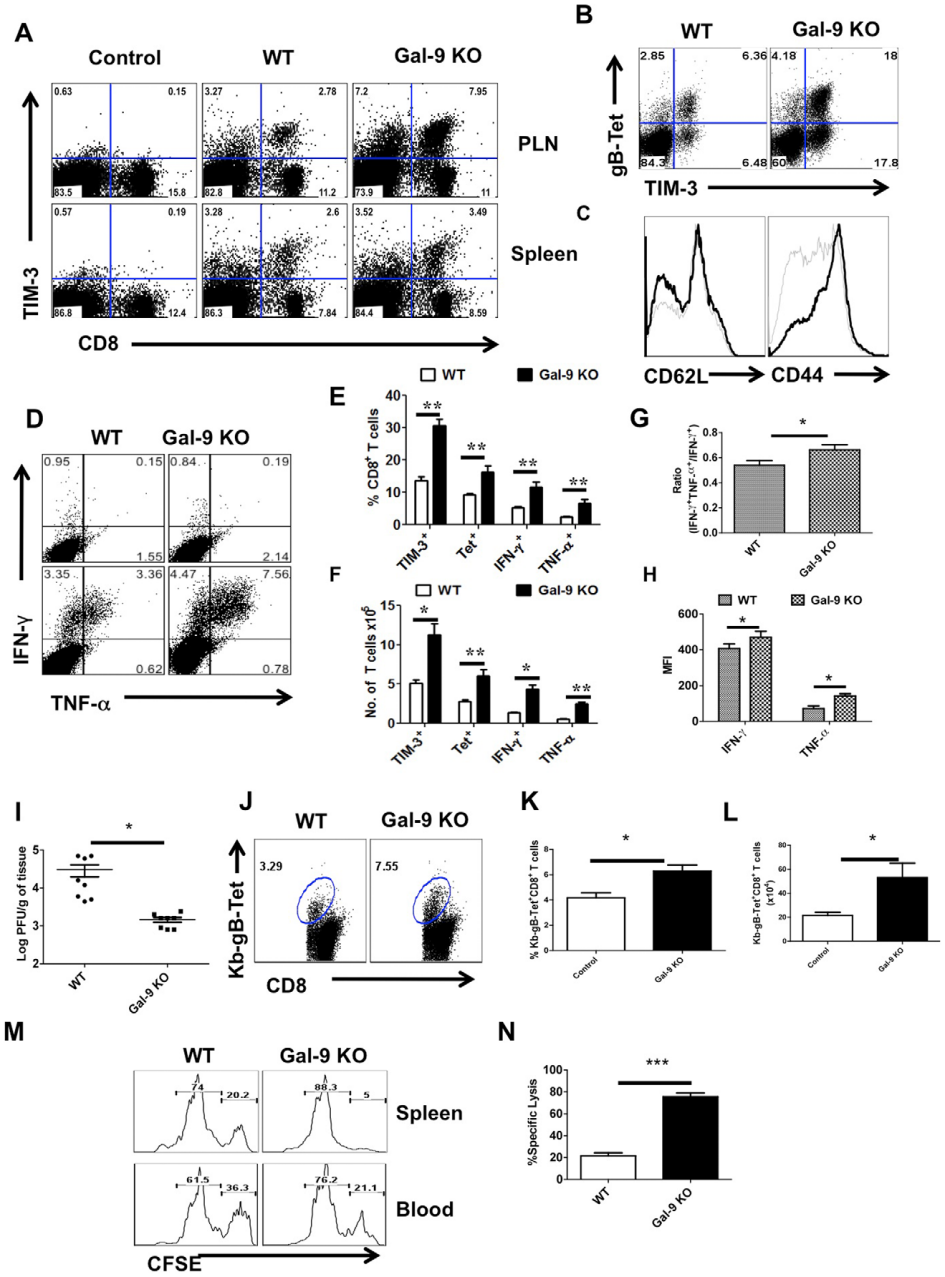
Galectin-9 knockout animals mount stronger virus-specific CD8^+^ T cell responses in the acute phase. Virus-specific CD8^+^ T cell responses were compared among age and gender matched HSV infected wild type (WT) and galectin-9 knockout (Gal-9 KO) animals at 5.5 dpi. The experiments were repeated three times with similar results. A. Representative FACS plots showing TIM-3 expression on CD8^+^ T cells isolated from the lymphoid organs of WT and Gal-9 KO animals. B. FACS plots showing the co-expression of TIM-3 and Kb-gB-Tet in WT and Gal-9 KO animals. C. Histograms show the expression of CD62L and CD44 on CD8^+^ T cells isolated from the PLNs of infected WT (thin lines) and Gal-9 KO (thick lines) animals. D. Representative FACS plots show the frequencies of SSIEFARL peptide-stimulated IFN-γ and TNF-α producing CD8^+^ T cells isolated from the PLNs of WT and Gal-9KO animals. E & F. The measurement of frequencies (E) and absolute numbers (F) of TIM-3^+^, Kb-gB-Tet^+^, IFN-γ and TNF-α producing CD8^+^ T cells isolated from the PLNs of infected WT (n = 6) and Gal-9 KO (n = 7) animals as described in A-D are shown. G. The bar diagram shows the ratios of SSIEFARL stimulated IFN-γ^+^TNF-α^+^ to IFN- γ^+^ CD8^+^ T cells isolated from PLNs of infected WT and Gal-9KO mice as depicted by FACS plots in D. H. The MFI of cytokines produced by CD8^+^ T cells is shown. I. Viral titers obtained from footpad tissues at 5.5 days pi of WT and Gal-9 KO are shown. J-N. Spleens of HSV infected WT and Gal-9 KO animals were analyzed for the measurement of tetramer^+^ CD8^+^ T cells (J-L) and their ability to kill peptide pulsed splenocytes (M and N) 11 dpi. Syngenic splenocytes were pulsed with SSIEFARL-peptide and labeled with high concentration (2.5 µM) of CFSE. As a control of antigen specificity, un-pulsed cells were labeled with low concentration (0.25 µM) of CFSE. 10^7^ cells of each type (CFSE^hi^ and CFSE^lo^) in 1∶1 ratio were transferred into in HSV infected WT and Gal-9 KO animals at 11 dpi. 2 hr post infusion, PBMCs and spleen cells were prepared from each animal and samples were acquired on FACS caliber and analysed by Flowjo software. The percentage of specific cell killing was determined as described in Materials and Methods section. The frequencies (J and K) and numbers (L) of Kb-gB-tet^+^ CD8^+^ T cells in the spleens of WT and Gal-9 KO animals at day 11 pi are shown. M. Representative FACS plots for the percentages of CFSE^hi^ and CFSE^lo^ cells are shown. N. The bar diagrams show the percent specific lysis in the spleens of recipients.

If indeed endogenous Gal-9 production acts to constrain the magnitude of the CD8^+^ T cell responses, then conceivably mice unable to produce Gal-9 due to gene knockout (KO) would respond with higher responses to HSV than WT animals. To evaluate this concept, age and gender matched WT and Gal-9 KO animals were infected via the footpads with HSV and the magnitude of CD8^+^ T cell responses were compared 5–6 days later (Fig 4). As shown at day 5.5 days pi, the frequencies (Fig 4A, B and E for Tet^+^ cells and Fig 4D and E for cytokine producing cells) and absolute numbers (Fig 4F) of virus-specific CD8^+^ T cells in the PLN as measured by tetramers and the ICCS assay, were increased up to 3 fold in the Gal-9 KO compared to WT mice. The expression levels of CD44 were higher and CD62L lower on CD8^+^ T cells from Gal-9 KO animals compared to WT (Fig 4C). This indicates that the more cells from KO animals were in activated state than those from WT animals. In experiments to compare the responses to HSV infection of Gal-9 and WT mice at different times pi, increased responsiveness of similar magnitude was observed at all times tested although there was a trend of greater differences after day 10dpi (Figure S3).

To provide evidence for a change in the quality of the CD8^+^ T cell response caused by the absence of Gal-9, we measured and compared mean fluorescence intensities (MFI) values for some cytokines as well as the proportions of responder T cells that produced two compared to a single cytokine. These properties have been advocated to indicate the presence of high quality T cells [18]. Our results demonstrate that in Gal-9 KO mice there was significantly increased frequencies (Fig 4D and E) and numbers (Fig 4F and G) of IFN-γ^ +^TNF-α^ +^ T cells, as well as a higher frequency of cells that had high MFI levels (Fig 4H) compared to WT mice. Additionally, IFN-γ^ +^IL-2^+^ T cell numbers were also increased in Gal-9 KO as compared to WT animals (Figure S4).

To measure the functional significance of the differential CD8^+^ T cell responses to HSV in Gal-9 KO and WT mice, in vivo cytotoxicity assays were performed after the peak time responses on 11 dpi. In these experiments animals received mixed transfers of 10^7^ CFSE labeled splenocytes that were pulsed with the MHC class I restricted SSIEFARL-peptide of HSV 1 (CFSE^hi^) along with 10^7^ un-pulsed cells (CFSE^lo^). To ensure the equal distribution in vivo of peptide pulsed and un-pulsed cells, the same numbers of both cell types were transferred into uninfected animals. After 2 hrs of transfer, spleens and blood samples were collected and analyzed by flow cytometry to measure differential killing of targets in WT and Gal-9 KO animals. Minimal killing of peptide pulsed splenocytes was seen in uninfected WT or the Gal-9 KO animals (data not shown). As is evident in Fig 4M and N, in the absence of Gal-9, CD8^+^ T cells were 3 to 4 times more effective at killing targets as compared to what occurred in WT animals. Accordingly, this indicates that endogenously produced Gal-9 may act to limit the effectiveness of virus-specific CD8^+^ T cell responses in vivo.

In other experiments, the ability of Gal-9 KO and WT animals were compared for their efficiency at clearing infectious virus from the infected footpad. As shown in Fig 4I, levels of virus in footpad homogenates measured at day 5.5 pi were 1.5 log lower on average in Gal-9 KO compared to samples from WT. Differences were also evident at other time points (data not shown). The results of both in vivo cytotoxicity and viral clearance experiments indicate that Gal-9 KO animals have more effective immunity to HSV.

### Galectin-9 Knockout animals mount stronger memory CD8^+^ T cell responses

**Figure 5:**
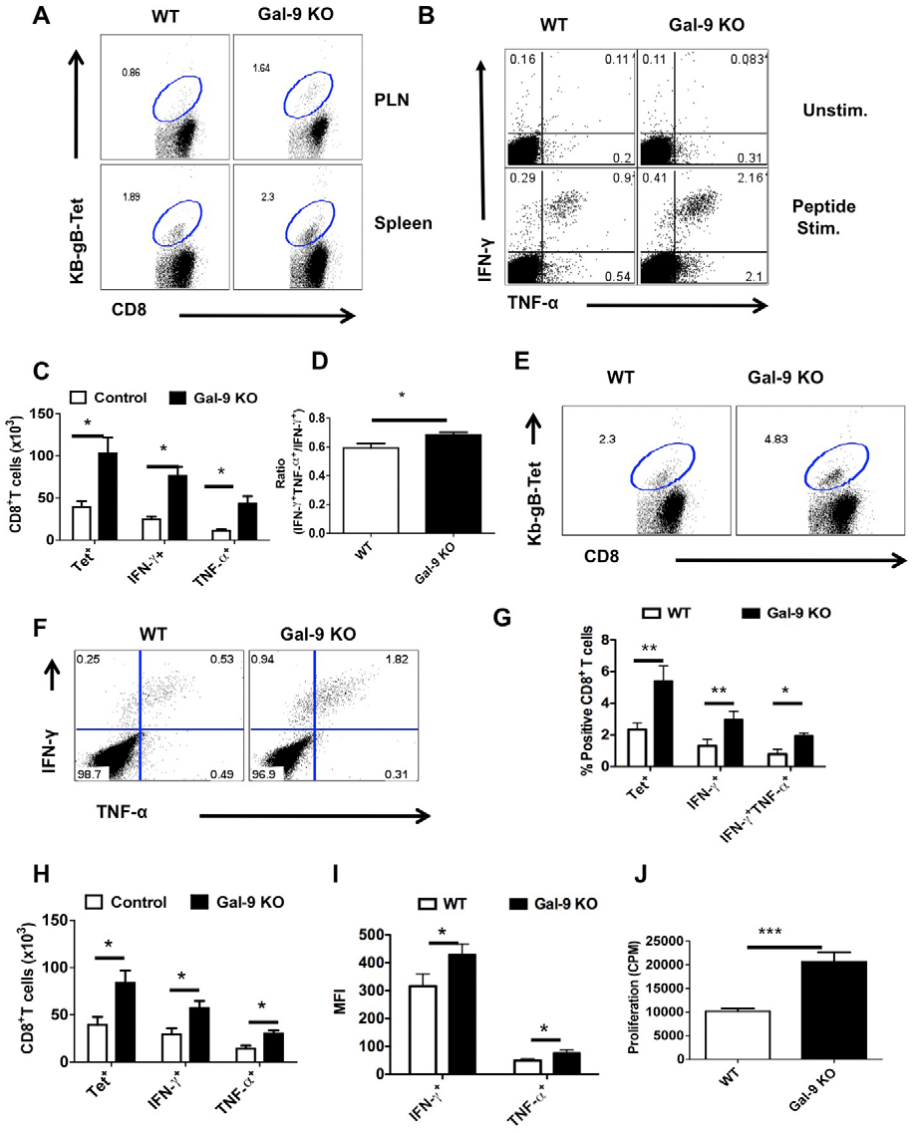
Galectin-9 KO animals develop sustained virus-specific CD8^+^ T cells memory responses. The lymphoid organs of HSV infected WT and Gal-9 KO animals were analyzed at day 32 or day 60 after infection for early and long-term memory responses using Kb-gB-tetramer and ICCS assays. All but long term memory experiments were repeated at least three times with four animals per group. A. Representative FACS plots showing the frequencies of Kb-gB-Tet^+^ cells in the spleens of WT and Gal-9 KO animals at 60 dpi. B. Representative FACS plots showing the frequencies of SSIEFARL peptide stimulated IFN-γ and TNF-α producing CD8^+^ T cells at 60 dpi. C. Bar diagram show the absolute numbers of Kb-gB-Tet^+^ and IFN-γ producing CD8^+^ T cells in the spleens of WT and Gal-9 KO animals as depicted by FACS plots in A and B at day 60. D. The bar diagram shows the ratio of SSIEFARL stimulated IFN-γ^+^TNF-α^+^ to IFN-γ^+^ CD8^+^ T cells isolated from WT and Gal-9 KO mice at 60 dpi. E-J. The animals previously infected with HSV for 32 days were re-infected with the same dose of HSV in footpads and the virus-specific CD8^+^ T cells responses were quantified in the PLN 2.5 dpi. E. Representative FACS plots show the frequencies of Kb-gB-Tet^+^ CD8^+^ T cells in the PLNs of WT (n = 4) and Gal-9 KO (n = 4) animals. F. Representative FACS plots show the frequencies of SSIFERAL-stimulated IFN-γ and TNF-α producing CD8^+^ T cell in the PLNs of WT (n = 4) and Gal-9 KO (n = 4) animals. G-H. The bar diagram shows the frequencies (G) and numbers (H) of Kb-gB-Tet^+^ and cytokines producing CD8^+^ T cells as depicted by FACS plots in E and F. I. MFI of cytokines produced by stimulated CD8^+^ T cell is shown. J. The peptide-specific proliferative response of PLNs cells isolated from re-infected animals at 2.5dpi is shown.

Comparisons of the magnitude and quality of HSV specific CD8^+^ T cell responses of Gal-9 KO and WT animals were also made 32 and 60 days pi to record memory phase effects. The results of a sample experiment, done at 60 days pi, are recorded in Fig 5. As in the acute phase, the memory responses of Gal-9 animals exceeded those of WT animals. Thus, the frequencies (Fig 5A) and total numbers (Fig 5C) of CD8^+^ and Kb-gB Tet^+^ T cells were increased by approximately 2.5 fold in Gal-9 KO, as compared to those of WT animals. In addition, the frequency and numbers of peptide-specific CD8^+^ T cells that produced both IFN-γ and TNF-α cytokines was also significantly higher in the spleens of Gal-9 KO population than in WT (Fig 5B-D) indicative of higher quality memory responses. Accordingly, in the absence of Gal-9, memory CD8^+^ T cell responses to HSV were increased.

Some WT and Gal-9 KO animals primed 32 days previously were re-infected in the footpad with HSV and the response in the PLN measured 2.5 days later by tetramers, ICCS and ex-vivo proliferative assays. Once again, the frequencies (Fig 5E and G) and numbers (Fig 5H) of Kb-gB-Tet^+^ and cytokine producing cells (Fig 5F-H) as well as peptide-specific proliferative responses (Fig 5J) were greater in Gal-9 KO as compared to WT animals. Although, the ratio of double vs single cytokine producers were not significantly different, the cells obtained from Gal-9 KO animals did show higher MFI for IFN-γ than those of WT animals (Fig 5I). Taken together the results of comparisons of the immune responses to HSV in Gal-9 KO and WT animals at the memory stage also revealed that Gal-9 might act in normal animals to limit the magnitude and efficiency of CD8^+^ T cell responses.

### How does Gal-9 influence CD8 responses in vivo?

**Figure 6:**
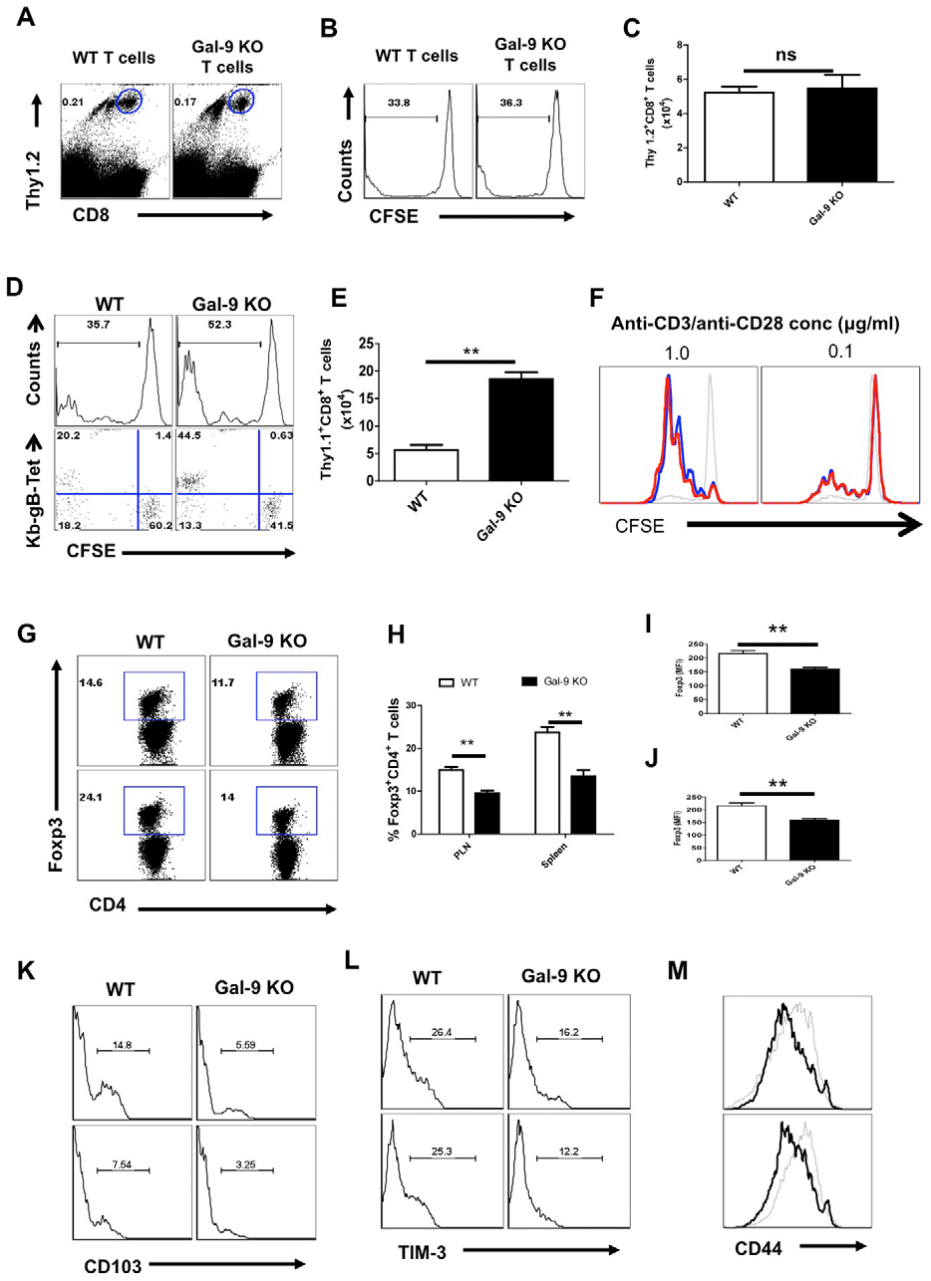
Galectin-9 deficiency extrinsically influences the virus-specific CD8^+^ T cell responses. Splenocytes (having 1×10^6^ of CD8^+^ T cells) isolated from either WT or Gal-9 KO animals after labeling with CFSE were transferred in WT Thy1.1 animals (n = 4 for each recipient) (A-C). Alternatively, CFSE labeled splenocytes (having 1×10^6^ of CD8^+^ T cells) isolated from Thy1.1 animals were transferred either in WT or in Gal-9 KO animals (D-E). The recipients were infected with 2.5×10^5^ HSV in footpad after 24 hr pi. Five dpi spleens and PLN cell suspensions were analyzed for the dilution of CFSE in transferred Thy1.2^+^ or Thy1.1^+^CD8^+^ T cells. A. FACS plots show the frequencies of transferred Thy1.2^+^ WT or the Gal-9 KO cells in the PLNs of Thy1.1 animals 5 dpi B. The representative histograms showing the extent of CFSE dilution in the transferred WT or the Gal-9 KO cells (gated on Thy1.2^+^CD8+ T cells as shown in A). C. Absolute numbers of the transferred Thy1.2^+^ CD8^+^ T cells WT or the Gal-9 KO cells in the PLNs of recipients. D. The extent of CFSE dilution in the transferred Thy1.1^+^CD8^+^ T cells isolated 5dpi from WT and Gal-9 KO animals. E. FACS plots show the co-expression of Kb-gB-Tet and CFSE in the dividing transferred Thy1.1^+^CD8^+^ T cells isolated from WT and Gal-9 KO animals. F. Purified CD8^+^ T cells (90% purity) from WT and Gal-9 KO animals after CFSE labeling were stimulated with plate bound anti-CD3 and CD28 mAb for 3 days and the extent of proliferation was measured by CFSE dilution in CD8^+^ T cells. Red lines show the CFSE staining in Gal-9 KO, Black thick lines show the CFSE staining in WT cells and black thin lines represent CFSE staining in un-stimulated cells. G-L. The lymphoid organs of infected WT and Gal-9 KO animals at 6 dpi were isolated and Foxp3^+^CD4^+^ T cells were characterized. G. FACS plots show the frequencies of Foxp3^+^ Tregs in PLNs (upper panel) and spleens (lower panel) of WT and Gal-9 KO. The percentages (H), MFI of Foxp3 expression on Foxp3^+^CD4^+^ Tregs isolated from PLN (I) and spleens (J) of WT and Gal-9 KO animals are shown. K-L. The frequencies of CD103^+^ (K) and TIM-3^+^ (L) Foxp3^+^ Tregs isolated from the draining PLNs (upper panel) and Spleens (lower panel) of WT and Gal-9 KO animals are shown. M. The expression of CD44 on Foxp3^+^CD4^+^ T cells isolated from the PLNs (upper panel) and spleens (lower panel) of WT (thin lines) and Gal-9 KO (thicker lines) animals.

Our observation that Gal-9 KO mice develop superior CD8^+^ T cell responses to HSV infection could be explained by the Gal-9 KO CD8^+^ T cells being intrinsically more responsive or being less inhibited when Gal-9 is absent in the environment. To further evaluate the situation, two types of experiments were done. In one approach, Thy1.2 naïve Gal-9 KO or WT CFSE labeled LN cells were transferred into Thy1.1 animals that were then infected via the footpad with HSV. In such experiments, no significant differences in the extent of proliferation by Gal-9 KO and WT CD8^+^ T cells could be observed (Fig 6A-C). This indicates that intrinsic reactivity differences between Gal-9 KO and WT T cells did not account for enhanced responses of Gal-9 KO animals.

In another approach, CFSE labeled naive splenocytes from Thy1.1 animals were transferred either into WT or Gal-9 KO animals that were subsequently infected via the footpad with HSV. Cell proliferation was measured by CFSE dilution in the donor cells and the total numbers of Kb-gB Tet^+^ donor cells that had accumulated in the PLN after 5 days were quantified. The donor cells proliferated to a similar degree in both recipient types (Fig 6D), but the accumulation of Kb-gB Tet^+^ specific cells 5 day later was approximately 4 fold higher in the Gal-9 KO than in WT animals (Fig 6E). In an additional in vitro experiment to compare the proliferative potential of Gal-9 KO and WT CD8^+^ T cells, CFSE labeled purified CD8^+^ T cells were stimulated with plate-bound anti-CD3 and anti-CD28 mAb for three days and the extent of CFSE dilution was measured. As shown in Fig 6F, both CD8^+^ T cell populations proliferated equally well. These ex-vivo and in-vivo results were consistent with the extrinsic environment accounting for the observed differences in the responsiveness of CD8^+^ T cells in Gal-9 KO and WT animals.

### Why does the environment of Gal-9 KO animals enhance HSV-specific CD8^+^ T cell responses?

A likely explanation for the better CD8^+^ T cell response to HSV of Gal-9 KO mice compared to WT mice is that the specific TIM-3^+^CD8^+^ T cell effectors will not undergo apoptosis in the absence of Gal-9. However, a contribution by the Foxp3+ regulatory T cell (Tregs) response could also be part of the explanation. In support of this we showed that compared to WT mice, Gal-9 KO animals had fewer Tregs (Fig 6G and H), especially those that were CD103^+^ (Fig 6K) and TIM-3^+^ (Fig 6L). Furthermore, the expression levels of Foxp3 (Fig 6G, I and J) and activation markers, such as CD103, TIM-3 and CD44, were lower on Tregs isolated from Gal-9 KO mice compared to those from WT animals (Fig 6K-M). Since, the CD8^+^ T cell response to HSV can be inhibited by Tregs [19], [20], having fewer of these cells, especially in the activated state, may also help explain why Gal-9 KO animals responded better than WT to HSV. That, Gal-9 KO mice have less natural Foxp3^+^ Tregs than WT animals after immunization with non-viral antigens has been reported by others [21].

### Reversal of Gal-9 inhibition of CD8^+^ T cell responses in vivo using α-lactose

**Figure 7:**
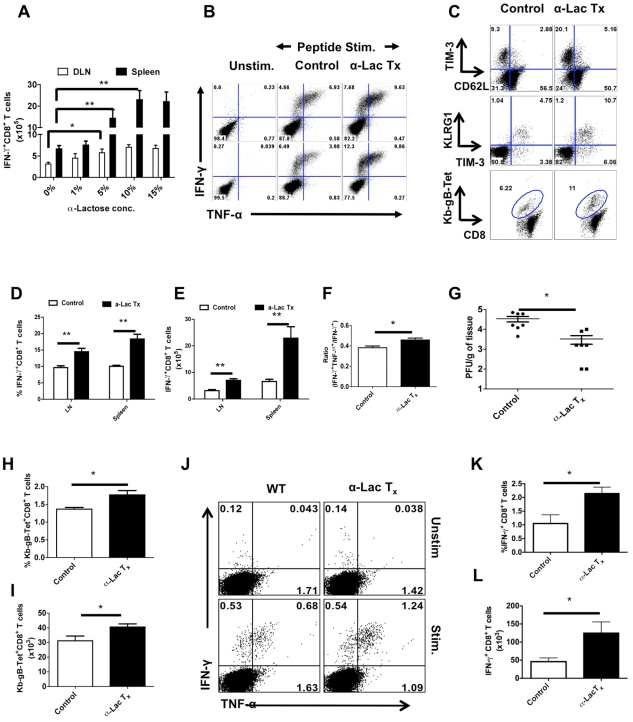
Administration of α-lactose solution in mice after HSV infection enhances the magnitude and quality of HSV-specific CD8^+^ T cells responses. C57B/6 animals were infected in each footpad with 2.5×10^5^ pfu of HSV and divided into groups. Animals were treated with different doses (27 mM, 137 mM, 277 mM, 416 mM) of α-lactose from day 3 until day 5 and those in the other group were given PBS. 12 hrs after last administration, animals were sacrificed and virus specific CD8^+^ T cell responses were quantified. A. Effect of different doses of α-lactose on the magnitude of virus-specific CD8^+^ T cells is shown. B. Representative FACS plots showing the frequencies of IFN-γ and TNF-α producing CD8^+^ T cells isolated from the PLN (upper panel) and spleen (lower panel) of α-lactose (277 mM) treated (n = 4) and control (n = 6) animals are shown. C. Expression of TIM-3 and CD62L (upper panel), TIM-3 and KLRG1 (middle panel) and Kb-gB-Tet on CD8^+^ T cells isolated from control and α-lactose treated animals are shown. D-E. Frequencies (D) and absolute numbers (E) of IFN-γ producing CD8^+^ T cells as depicted by FACS plots in B are shown. F. The bar diagram shows the ratio of SSIEFARL stimulated IFN-γ^+^TNF-α^+^ to IFN-γ^+^ CD8^+^ T cell. G. Viral titers in the footpad tissues of α-lactose treated and control animals are shown. H-J. HSV infected animals were given 277 mM α-lactose ip day 1 until day 7 and their CD8^+^ T cell responses were compared with that of control animals at 30 dpi. The frequencies (H) and numbers (I) of Kb-gB-Tet^+^CD8^+^ T cells are shown. J-L. The frequencies (J and K) and absolute numbers (L) of SSIEFARL stimulated IFN-γ and TNF-α producing CD8^+^ T cells isolated form control (n = 4) and α-lactose treated (n = 4) animals are shown.

Further evidence that endogenously produced Gal-9 may be acting in vivo to limit the extent of antiviral CD8^+^ T cell responses was obtained by infusing an excess of the sugar that binds to Gal-9 and which interferes with its binding to the TIM-3 receptor. To this end, HSV infected animals were infused ip twice daily with 200 µl of α-lactose solutions (27 mM, 137 mM, 277 mM, 416 mM) starting from day 3 until day 5. Subsequently, virus-specific CD8^+^ T cell responses were quantified 12 hr later and compared to untreated controls. As shown in Fig 7A-F, mice infused with 277 mM α**-**lactose had significantly enhanced and higher quality CD8^+^ T cells responses than untreated controls. The effect was dose dependant with the 277 mM lactose solution giving the maximal effect (Fig 7A). With this dose, the numbers of gB_498–505_ peptide stimulated IFN-γ producing CD8^+^ T cells at peak response times were increased 2–3 fold in the PLN and >3 fold in the spleen compared to control animals (Fig 7B, D and E). A similar pattern of results was revealed using Kb-gB Tet^+^, TIM-3 and KLRG-1 to measure results (Fig 7C). Interestingly, the α-lactose recipients also had a small but significantly higher ratio of double:single cytokine producing cells indicative of better quality responses (Fig 7F). We also quantified the viral loads in the footpads of HSV infected animals that were either treated with α**-**lactose or PBS alone. Viral loads were less (p≤0.05) in α-lactose treated animals at day 6 pi than in controls (Fig 7G).

Virus-specific CD8^+^ T cell responses were also compared at 32 days pi in α-lactose treated and control animals in limited studies. A small, but significant increase in the frequencies (Fig 7H) as well as numbers (Fig 7I) of Kb-gB Tet^+^T cells, IFN-γ^+^ and TNFα^+^ -producing CD8^+^ T cells (Fig 7J-L) was observed in the α-lactose treated animals. No significant changes in the number of double cytokine-producing cells between groups were detected.

**Figure 8:**
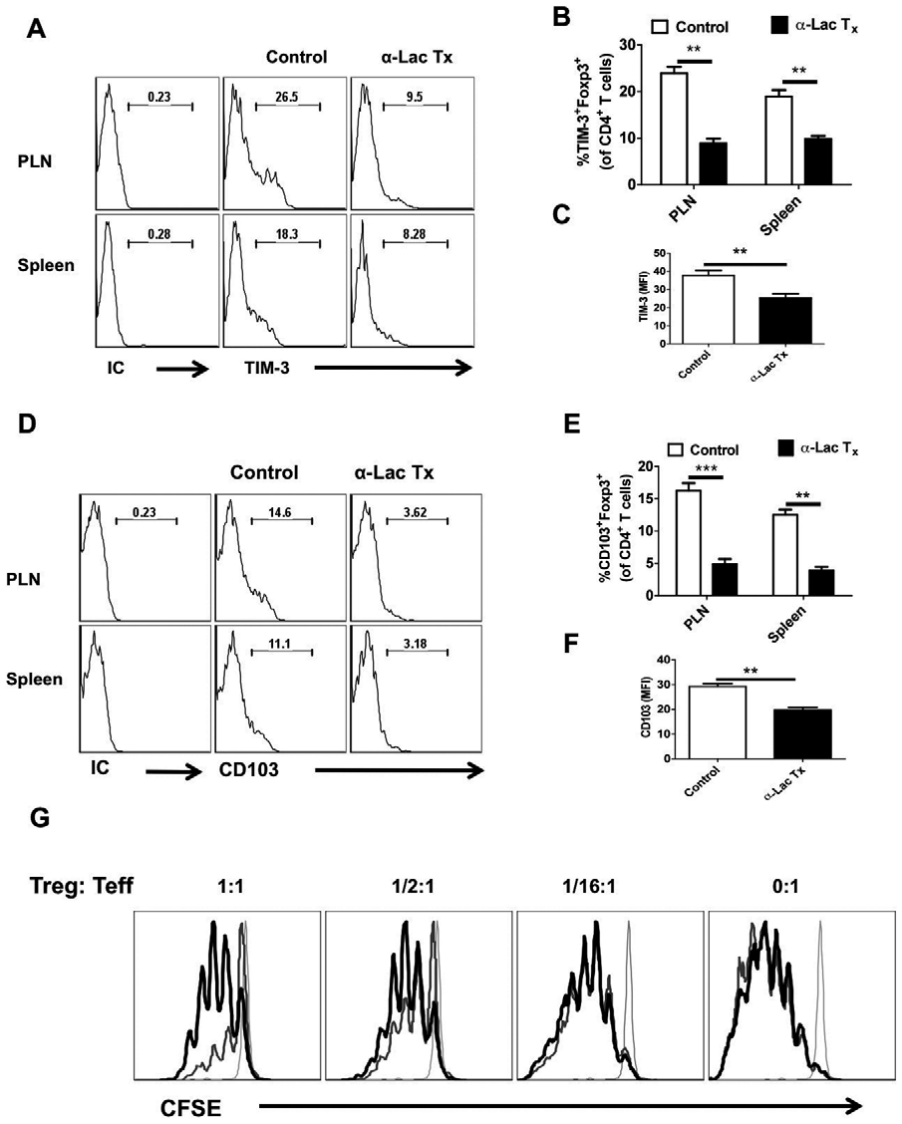
Administration of α-lactose diminishes regulatory T cells responses. LN cells and splenocytes isolated from HSV infected animals that were either treated with α-lactose or controls as described in Fig 4, were analyzed for the expression of activation markers and activity. A-C. FACS plots depicting the expression (% positive (A and B) and MFI (C) of TIM-3 on gated CD4^+^Foxp3^+^ T cells in the control and α-lactose treated animals are shown. D-F. FACS plots showing the expression (% positive (D and E) and MFI (F) of CD103 on gated CD4^+^Foxp3^+^ T cells in the control and α-lactose treated animals are shown. G. The suppressive activity of Tregs is reduced after α-lactose treatment. Foxp3-GFP Knock-in animals were infected with HSV and divided into two groups. One group was treated with α-lactose (277mM solution) and the other group of animals was left untreated. GFP^+^ cells were sorted after 6 days of HSV infection and co-cultured with CFSE labeled Thy1.1 CD4^+^CD25- cells and T cell depleted splenocytes isolated from uninfected animals Thy1.1 B/6 animals. FACS plots (G) show the extent of dilution of CFSE as an indicator of proliferation in Thy1.1^+^CD4^+^and its inhibition in the presence of Tregs. CFSE staining in un-stimulated cells (faint line), stimulated cells in the presence of Tregs isolated from control animals (thinner line) and stimulated cells in the presence of Tregs isolated from α-lactose treated animals (thickest line) are shown.

We also measured the phenotype and function of the Foxp3^+^ Treg isolated from control and α**-**lactose treated animals at day 5.5 pi (Fig 8A-F). The mean fluorescence intensities of Foxp3, CD103 and TIM-3 expression were decreased in Treg isolated from α**-**lactose treated animals as compared to control animals (Fig 8C and F). Additionally, the frequencies and the total number per PLN of CD103^+^ (Fig 8A and B) and TIM-3^+^ (Fig 8D and E) Foxp3^+^CD4^+^ T cells was also reduced in α**-**lactose treated animals as compared to controls. Furthermore, the in vitro suppressive activity of Foxp3^+^CD4^+^ T cells isolated from α**-**lactose treated animals was also reduced compared to controls when compared by in vitro suppression assays. These data indicate that inhibition of Gal-9 binding resulting in a decline of Treg function (Fig 8G).

Taken together, our results with α-lactose infusion to block Gal-9 activity support the concept that endogenously produced Gal-9 acts to reduce the magnitude and function of anti-HSV CD8^+^ T cell responses. The effect was in part mediated by effects on Treg responses.

### Gal-9 administration diminishes the HSV-specific CD8^+^ T cell response and delays viral clearance

**Figure 9:**
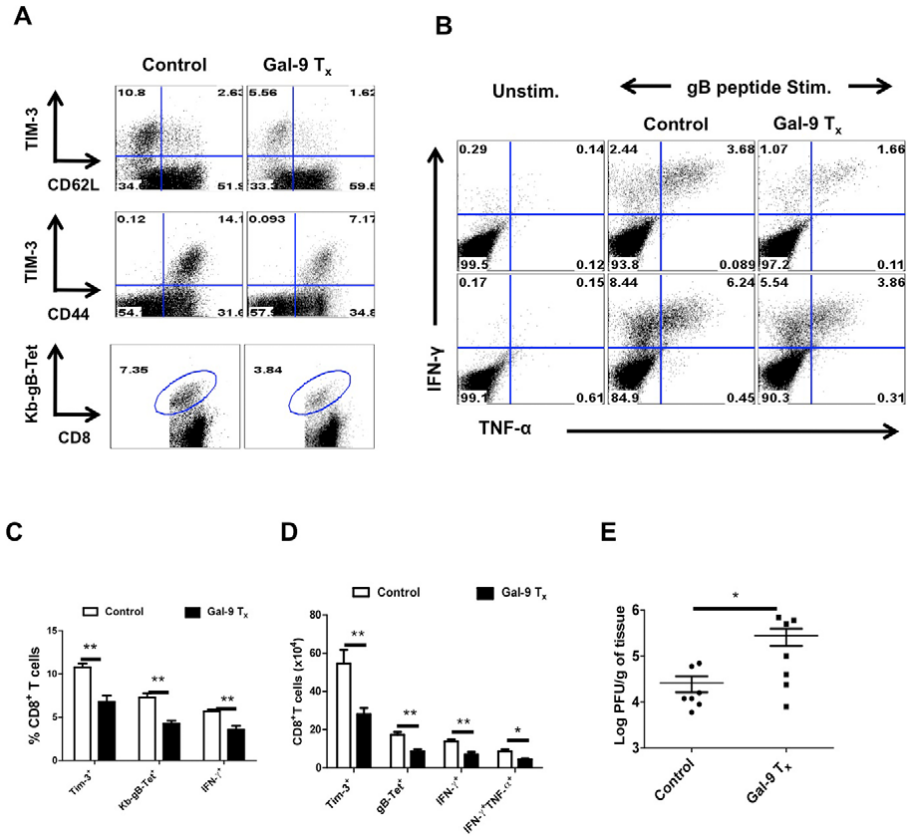
Administration of galectin-9 during the expansion phase after HSV infection diminishes the magnitude of virus-specific CD8^+^ T cell responses. C57B/6 animals (n = 10) were infected in each footpad with 2.5×10^5^ PFU of HSV and divided into two groups. Animals in one of the groups were treated with 125 µg of Gal-9 from day 3 until day 5 and those in the other group were given PBS. 12 hr after last injection, animals were sacrificed and single cell suspensions of their PLNs and spleens were analyzed flow cytometrically for virus specific CD8^+^ T cell responses (surface staining and SSIEFARL peptide stimulated intracellular IFN-γ and TNF-α producing CD8 T cells). A. Representative FACS plots for surface staining of CD8^+^ T cells isolated from the PLNs for the expression of TIM-3, CD44, CD62L and Kb-gB-Tet^+^ in Gal-9 treated and control animals are shown. B. Representative FACS plots for the frequencies of peptide stimulated IFN-γ and TNF-α producing CD8^+^ T cells in the PLN (upper panel) and spleens (lower panel) are shown. C-D. Frequencies (C) as well as absolute numbers (D) of CD8^+^ T cells positive for TIM-3, KB-gb-Tet, IFN-γ and TNF-α isolated from PLNs of control and treated mice are shown. E. The viral titers in the footpads of control and treated animals 12 hrs after last injection as quantified by standard plaque assays are shown. The experiments were performed two times with 5 animals per group each time.

The previous studies all relate to the influence of endogenous Gal-9 binding to the TIM-3 receptor on virus reactive CD8^+^ T cells. In this experiment, we measured the outcome of adding exogenous Gal-9 on the magnitude of the anti-HSV CD8^+^ T cell response in HSV infected WT mice. Animals were given 125 µg ip of Gal-9 during the expansion phase (from day 3 to day 5 pi) and virus-specific CD8^+^ T cell responses were compared with untreated animals 12 hrs after the last injection. As shown in Fig 9A-D, the recipients of Gal-9 had significantly reduced frequencies as well as 2.5 fold reductions in total numbers per PLN of Kb-gB Tet^+^ as well as TIM-3^+^ CD8^+^ T cells. Furthermore, Gal-9 treated animals had significantly higher viral loads (1–1.5 logs) in their footpads compared to controls (Fig 9E).

These experiments demonstrate that exogenous Gal-9 administration reduces the efficiency of CD8^+^ T cell mediated immunity to HSV infection.

## Discussion

This communication investigates the influence of galectin-9 binding to its receptor TIM-3 on the size and quality of CD8^+^ T cell mediated immunity to a virus infection. We demonstrate that Gal-9 acts to constrain CD8^+^ T cell immunity to HSV infection. In support of this, we show that animals unable to produce Gal-9, because of gene knockout, develop acute and memory responses to HSV that are of greater magnitude and better quality than those that occur in normal infected animals. Interestingly, infusion of normal infected mice with α-lactose, the sugar that binds to the carbohydrate-binding domain of Gal-9 limiting its TIM-3 receptor engagement [9], also caused a more elevated and higher quality CD8^+^ T cell response to HSV. Such sugar treated infected mice also had expanded populations of memory CD8^+^ T cells. The mechanisms responsible for the outcome of the Gal-9/TIM-3 interaction in normal infected mice involved both inhibitory effects on TIM-3^+^ effector cells, as well as the promotion of Foxp3^+^ regulatory T cell activity. Our results indicate that manipulating galectin signals, as can be achieved using appropriate sugars, may represent a convenient and inexpensive approach to enhance acute and memory responses to infectious agents.

Our observation that negating signals delivered by Gal-9 resulted in elevated CD8^+^ T cell responses to HSV came as a surprise. Thus, the expansion of virus specific CD8^+^ T cells in Gal-9 KO mice in the acute response was as much as four fold greater than occurred in WT animals. We could show that effect was extrinsically mediated by the presence of Gal-9 during the development of the immune response. Accordingly, Gal-9 KO and WT CD8^+^ T cells responded equally when transferred into the virus infected neutral environment. In contrast, WT cells transferred to a Gal-9 deficient environment responded better to HSV than occurred when the same cell population was transferred to mice that could produce Gal-9 normally. Thus, our experiments support the idea that Gal-9, presumably by its binding to the TIM-3 receptor, serves to minimize the expansion of the CD8^+^ T effector response to HSV.

Further support for a constraining influence of Gal-9 came from novel observations in WT mice showing that administering the sugar α-lactose systemically for three days after HSV infection to normal mice also resulted in enhanced HSV specific CD8^+^ T cell responses compared to controls. In fact, the response pattern was similar to that observed in Gal-9 KO animals. Since α-lactose binds to the carbohydrate-binding domain of Gal-9 [9], it presumably acted by blunting the interaction of the endogenous Gal-9 to its TIM-3 receptors. In our studies, α-lactose was administered on multiple occasions intraperitoneally, but it will be of interest to determine if the lactose enhancement effect can also be achieved by an administration procedure that be would be practical for use with vaccines. This issue is currently being investigated in our laboratory.

In both Gal-9 KO and sugar inhibited virus infected WT mice, the consequence of blunting Gal-9 signals was not only an increase in the magnitude of the HSV-specific CD8^+^ T cell response, but also a change in its quality. The latter conclusion came from the observation that the frequency of gB peptide specific T cells that produced multiple cytokines, as well as high cytokine producing cells, was significantly higher in Gal-9 blunted animals compared to controls (particularly in the acute phase of the response). Multiple cytokine production, as well as the presence of high cytokine producing effector T cells, is commonly used as an indicator of more functional T cells [18], although formally showing the functional value of this increased quality in vivo is rarely done. In our studies, we did show that mice with blunted Gal-9 responses cleared virus more effectively, but did not establish if the better control was explained by quantitative or qualitative changes of CD8^+^ T cell activity, or perhaps was caused by effects on other aspects of immune defense. Whatever the explanation, the observation that blunting reactivity to Gal-9 results in improved immunity requires comment since it seems counterintuitive in terms of protecting the host against a virus infection. We advocate that the phenomenon may relate to the needs of the host to minimize collateral tissue damage that is a common occurrence especially during chronic T cell responses to foreign antigens [22], [23]. In support of this, reports show that the Gal-9 signals to TIM-3 help to minimize the severity of autoimmune lesions [7], [24]. For example, Gal-9 KO mice develop more severe lesions of collagen-induced arthritis than normal animals [21]. We have also observed that Gal-9 KO mice are more susceptible than WT animals to the immunopathological disease herpetic stromal keratitis (SK) (unpublished results). Using this model, which is a HSV induced lesion in the eye orchestrated by CD4^+^ T cells [25], we have shown that Gal-9/TIM-3 engagement plays a beneficial role. Accordingly, administering exogenous Gal-9 locally in infected mice minimizes the severity of SK lesions and can even cause their resolution [8]. Thus, our observation that the interaction of endogenously produced Gal-9 with its TIM-3 receptor can be detrimental to the efficiency of protective CD8^+^ T cell mediated immunity represents a modest dispensation by the body to avoid exuberant damaging reactions.

With regard to the mechanisms by which Gal-9 acts to counter the extent of the CD8^+^ T cell response, several events could be occurring. Most likely, however, the principal mechanism involves the induction of apoptosis in TIM-3 positive effector cells as we could demonstrate in vitro. Others too have reported that Gal-9 can cause the apoptosis of some, although not all, subsets of TIM-3 expressing T cells [8], [26]. Curiously, as we reported recently, TIM-3^+^Foxp3^+^ Tregs do not undergo Gal-9 induced apoptosis for reasons that are yet to be ascertained. Furthermore, Gal-9 administration into antigen stimulated mice may induce expansion of the Foxp3^+^ T cell population likely by causing some conventional T cells to become Foxp3^+^ and regulatory in function [8], [21]. In fact, one effect of blunting Gal-9 observed in the present investigations was an expansion of Treg numbers and function. Thus, lower expression levels of several activation and functional markers was evident on Foxp3^+^ Treg isolated from Gal-9 KO compared to WT infected animals. Similarly, blunting Gal-9 function with α-lactose produced a similar change in Treg numbers and phenotype as observed in Gal-9 KO mice. Furthermore, Foxp3^+^ cells isolated from HSV infected Foxp3-GFP-knock-in mice had lower in vitro suppressive activity when taken from α-lactose treated than untreated animals.

Since, as we and others have shown previously [19], Treg limit the magnitude of T cell responses to virus infections, part of the effect caused by blunting Gal-9 signals could be explained by diminished Treg activity. It is also conceivable that the Treg involved in limiting the magnitude of CD8^+^ T cell responses could have a distinct phenotype. Thus it was shown recently that Treg might acquire a genetic program similar to that of the cell type being suppressed and that unique sets of Treg may be responsible for controlling different components of immunity [27], [28], [29]. Our observations that TIM-3 is up-regulated on Tregs after HSV infection and that the increased CD8^+^ T cell responses were associated with diminished numbers of TIM-3^+^ regulatory T cells in animals with blunted Gal-9 activity, could also mean that Tregs that are TIM-3^+^ are preferentially involved in controlling CD8^+^ T cell responses. Additional studies are underway to establish the relative effects of Gal-9 on effectors and regulators, as well as to further identify the phenotype of the Tregs involved in constraining HSV specific CD8^+^ T cell activity.

Apart from direct effects of Gal-9 on TIM-3^+^ T cells, Gal-9 may also influence some functions of the innate immune system. Reported effects include enhanced chemotaxis and activation of granulocytes as well as the maturation of dendritic cells. The latter effect of Gal-9 was shown to be responsible for an improved anti-tumor CD8^+^ T cell response [30]. We doubt if effects on DCs explained our findings since populations isolated 24 hrs from the PLNs after footpad infection of Gal-9 KO mice showed no significant differences in phenotypic and functional markers than cells from WT animals (data not shown). However, further investigation on the influence of galectins on components of innate immune is currently underway in our laboratory.

Perhaps the most important observation we made was that animals with blunted Gal-9 signals (Gal-9 KO as well as α-lactose treated) had expanded populations of memory CD8^+^ T cells. At two months after infection, populations were approximately two-fold higher. Currently, we cannot explain why more CD8^+^ T cells survive to become memory cells in the absence of Gal-9. Further studies are underway to determine if the Gal-9 influence is expressed predominantly during the induction, contraction or maintenance phases of T cell mediated immune responses, as well as to explore if modulating Gal-9 levels can influence an already established pattern of responsiveness. However, an exciting potential application of our findings is that memory CD8^+^ T cell populations can be expanded using simple sugars. This could represent an easy, non-toxic and inexpensive way to enhance anti-microbial immunity. Accordingly, an expanded memory population may cause the outcome of re-infection or reactivation by a pathogen to be subclinical rather than disease producing. Currently, we know little about the influence of sugars on the efficiency of innate and adaptive immunity. This issue merits more investigation.

## Supporting Information

Figure S1 C57BL/6 animals were infected in each hind footpad with 2.5x10^5^ PFU of HSV. At different time points after infection, splenocytes isolated from three animals at each time point were analyzed flow cytometrically for TIM-3 and IFN-γ expression. A. Representative FACS plots show the frequencies of TIM-3^+^ and IFN-γ^+^ in CD8^+^ T cell gated populations. B. Frequencies of TIM-3^+^ and IFN-γ^+^ CD8^+^ T cells are shown.
(0.25 MB TIF)

Figure S2 C57BL/6 animals were infected in each hind footpad with 2.5x10^5^ PFU of HSV and the concentration of Gal-9 in the PLN samples of infected animals was measured by sandwich ELISA using anti-Gal-9 (108A2) mAb.
(0.18 MB TIF)

Figure S3 Comparison of antigen-specific CD8^+^ T cell responses (measured by ICCS assay) in Gal-9 KO and WT mice at different times after HSV infection. Age and gender matched WT and Gal-9 KO animals were infected in footpad with 2.5x10^5^ PFU of HSV KOS. At indicated time points, three animals were sacrificed and the SSIEFERAL specific IFN-γ producing cell numbers were quantified by ICCS assays. Figure shows the mean of three observations at each time point.
(0.10 MB TIF)

Figure S4 C57BL/6 WT and Gal-9 KO animals were infected in each hind footpad with 2.5x10^5^ PFU of HSV and cytokine producing CD8^+^ T cells were quantified using ICCS assays at 5.5dpi. The FACS plots (A) and bar diagram (B) show the frequencies of SSIEFARL peptide stimulated IL-2 and IFN-γ producing CD8^+^ T cells.
(0.27 MB TIF)
