# Supplementary figures and images for: Galectin-9/TIM-3 Interaction Regulates Virus-Specific Primary and Memory CD8+ T Cell Response
Source: PLoS Pathog. 2010 May 6;6(5):e1000882. doi: 10.1371/journal.ppat.1000882 (PMC2865527; doi:10.1371/journal.ppat.1000882)

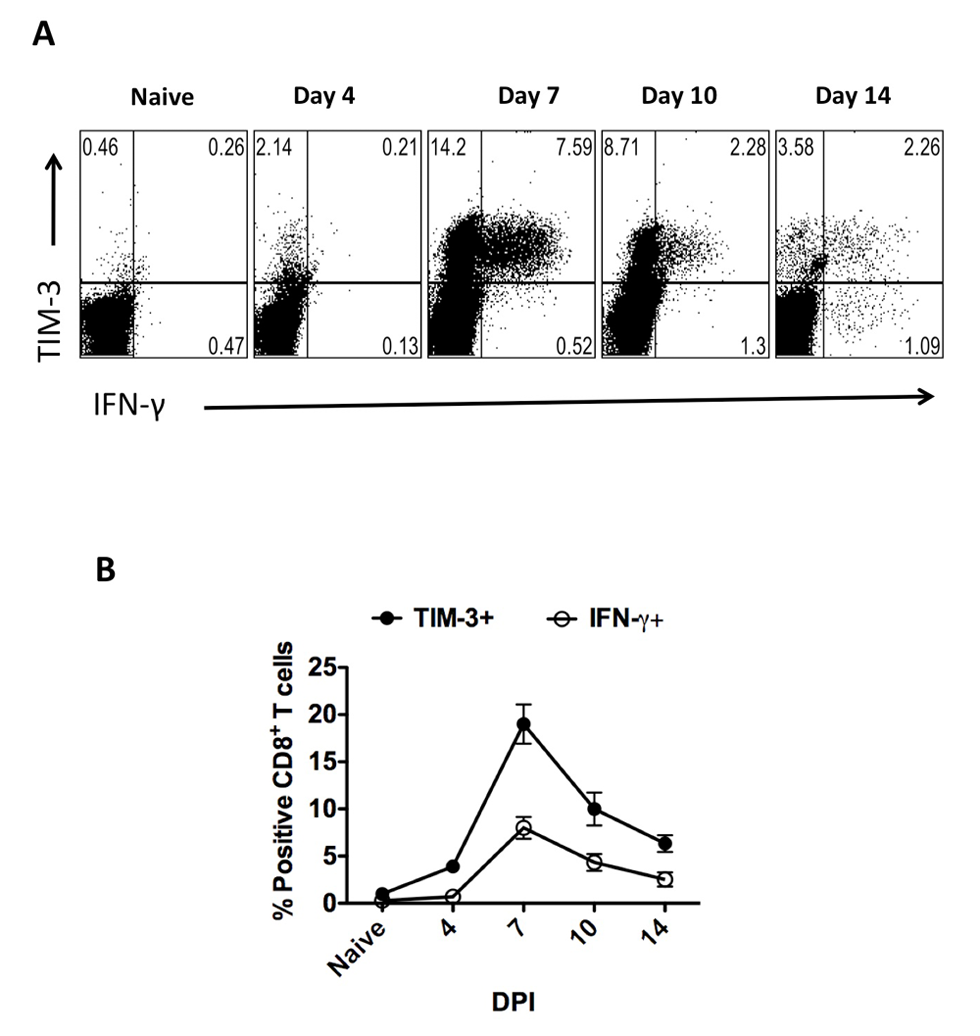

Supplement: Figure S1 — C57BL/6 animals were infected in each hind footpad with 2.5x10^5^ PFU of HSV. At different time points after infection, splenocytes isolated from three animals at each time point were analyzed flow cytometrically for TIM-3 and IFN-γ expression. A. Representative FACS plots show the frequencies of TIM-3+ and IFN-γ+ in CD8+ T cell gated populations. B. Frequencies of TIM-3+ and IFN-γ+ CD8+ T cells are shown. (0.25 MB TIF) [file ppat.1000882.s001.tif]

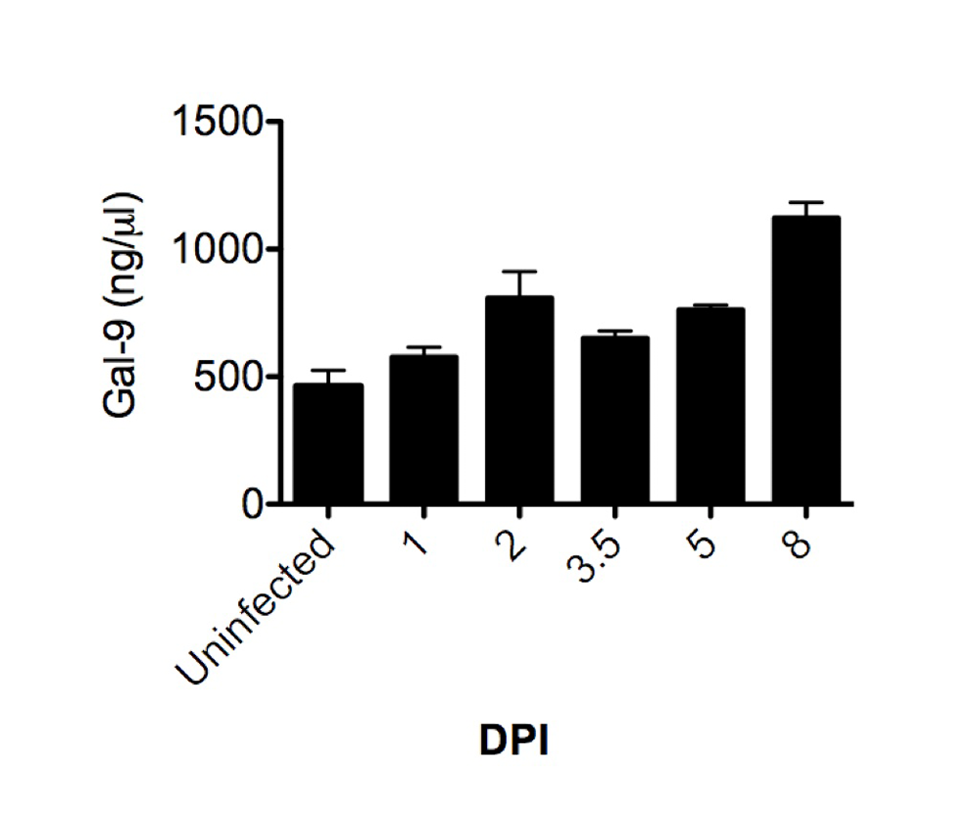

Supplement: Figure S2 — C57BL/6 animals were infected in each hind footpad with 2.5x10^5^ PFU of HSV and the concentration of Gal-9 in the PLN samples of infected animals was measured by sandwich ELISA using anti-Gal-9 (108A2) mAb. (0.18 MB TIF) [file ppat.1000882.s002.tif]

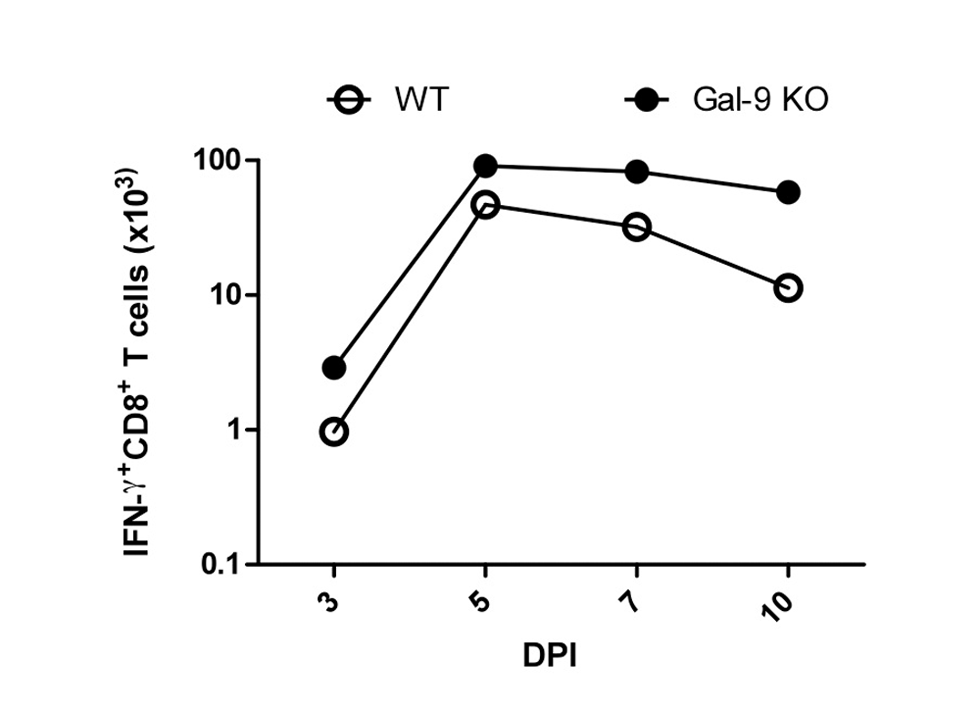

Supplement: Figure S3 — Comparison of antigen-specific CD8+ T cell responses (measured by ICCS assay) in Gal-9 KO and WT mice at different times after HSV infection. Age and gender matched WT and Gal-9 KO animals were infected in footpad with 2.5x10^5^ PFU of HSV KOS. At indicated time points, three animals were sacrificed and the SSIEFERAL specific IFN-γ producing cell numbers were quantified by ICCS assays. Figure shows the mean of three observations at each time point. (0.10 MB TIF) [file ppat.1000882.s003.tif]

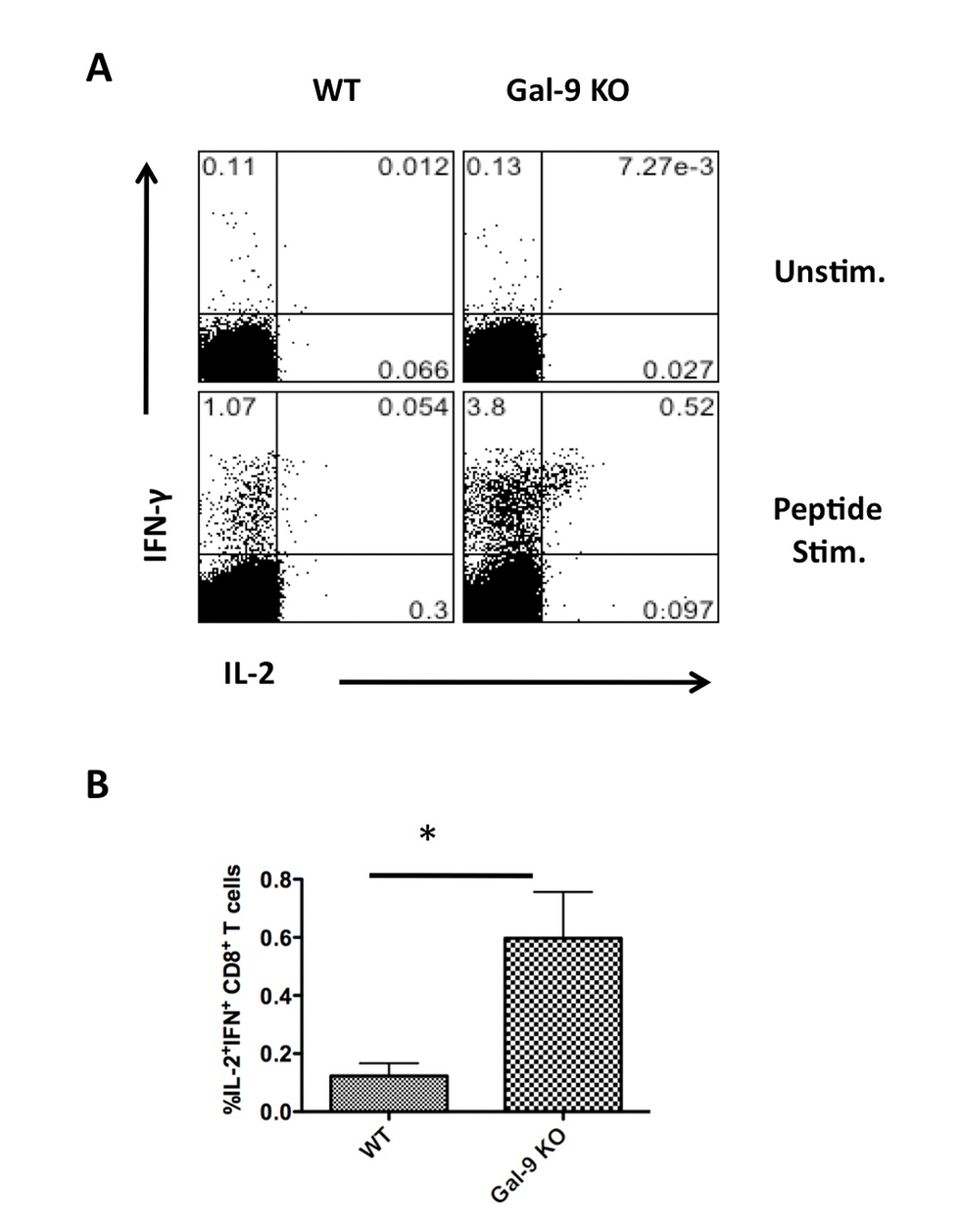

Supplement: Figure S4 — C57BL/6 WT and Gal-9 KO animals were infected in each hind footpad with 2.5x10^5^ PFU of HSV and cytokine producing CD8+ T cells were quantified using ICCS assays at 5.5dpi. The FACS plots (A) and bar diagram (B) show the frequencies of SSIEFARL peptide stimulated IL-2 and IFN-γ producing CD8+ T cells. (0.27 MB TIF) [file ppat.1000882.s004.tif]
